# Neutrophil‐to‐lymphocyte ratio for risk stratification in acute myocarditis across the left ventricular ejection fraction spectrum

**DOI:** 10.1002/ejhf.70072

**Published:** 2025-10-16

**Authors:** Cristina Madaudo, Amitai Segev, Emanuele Bobbio, Chiara Baggio, Jonathan Schütze, Piero Gentile, Marta Sanguineti, Luca Monzo, Matteo Schettino, Emma Ferone, Ahmed Elsanhoury, Anan Younis, Matteo Palazzini, Adriana Ferroni, Valentina Giani, Matthew Sadler, Mohammad Albarjas, Leonardo Calò, Christian Lars Polte, Andrea Garascia, Stefano Figliozzi, Paul A. Scott, Ajay M. Shah, Alfredo Ruggero Galassi, Mauro Giacca, Gianfranco Sinagra, Entela Bollano, Theresa McDonagh, Carsten Tschöpe, Giuseppina Novo, Enrico Ammirati, Roy Beigel, Christoph Gräni, Marco Merlo, Pietro Ameri, Antonio Cannata, Daniel I. Bromage, CAIR Collaboration

**Affiliations:** ^1^ King's College London British Heart Foundation Centre of Excellence School of Cardiovascular Medicine & Sciences London UK; ^2^ Department of Health Promotion Mother and Child Care, Internal Medicine and Medical Specialties (ProMISE) University of Palermo Palermo Italy; ^3^ Cardiovascular Division Chaim Sheba Medical Center, Tel Hashomer. The Faculty of Medicine, Tel‐Aviv University Ramat‐Gan Israel; ^4^ Department of Cardiology, Sahlgrenska University Hospital Gothenburg Sweden; ^5^ Department of Molecular and Clinical Medicine, Institute of Medicine at Sahlgrenska Academy University of Gothenburg Gothenburg Sweden; ^6^ CardioThoracoVascular Department, Azienda Sanitaria Universitaria Giuliano‐Isontina Trieste Italy; ^7^ Department of Cardiology, Inselspital, Bern University Hospital University of Bern Bern Switzerland; ^8^ ASST Grande Ospedale Metropolitano Niguarda Milan Italy; ^9^ Department of Internal Medicine University of Genova Genoa Italy; ^10^ Policlinico Casilino Rome Italy; ^11^ Université de Lorraine, Centre d'Investigations Cliniques Plurithématique 1433 and Inserm U1116, CHRU Nancy, FCRIN INI‐CRCT (Cardiovascular and Renal Clinical Trialists) Nancy France; ^12^ Berlin Institute of Health (BIH) Center for Regenerative Therapies (BCRT) Berlin Germany; ^13^ Department of Cardiology, Angiology, and Intensive Medicine (CVK), German Heart Center at Charite (DHZC) Berlin Germany; ^14^ German Centre for Cardiovascular Research (DZHK), partner site Berlin Berlin Germany; ^15^ King's College Hospital NHS Foundation Trust London UK; ^16^ Princess Royal University Hospital, Orpington London UK; ^17^ Department of Clinical Physiology and Radiology, Sahlgrenska University Hospital Gothenburg Sweden; ^18^ Department of Biomedical Sciences Humanitas University Pieve Emanuele Italy; ^19^ IRCCS Humanitas Research Hospital Rozzano Italy; ^20^ Institute of Heart Diseases Wroclaw Medical University Wroclaw Poland; ^21^ Cardiac, Thoracic and Vascular Department, IRCCS Ospedale Policlinico San Martino Genoa Italy

**Keywords:** Myocarditis, Neutrophil, Lymphocyte, Neutrophil‐to‐lymphocyte ratio, Outcomes, Risk prediction

## Abstract

**Aims:**

Acute myocarditis (AM) is a heterogeneous clinical condition. Several classification models have been proposed to predict adverse clinical outcomes, but risk stratification remains challenging, particularly for patients presenting with preserved left ventricular ejection fraction (LVEF). Neutrophil‐to‐lymphocyte ratio (NLR) is a useful tool for risk stratification in patients with AM. This study aimed to compare the predictive accuracy of available risk stratification models, including NLR, for identifying patients with AM at increased risk of adverse events.

**Methods and results:**

The study included 1150 patients with biopsy‐ or cardiac magnetic resonance (CMR)‐proven AM from 10 hospitals in six countries. Baseline clinical, laboratory, echocardiographic and CMR data, and clinical outcomes, were collected. The population was divided into four groups based on published models of high‐risk AM (complicated, fulminant, high‐risk, and NLR ≥4). The primary outcome was all‐cause mortality and heart transplantation. During a median follow‐up of 228 weeks (interquartile range 114–339), 63 events occurred in 60 patients (5.2%) who experienced the primary outcome. NLR (area under the curve [AUC] 0.72) performed similarly to complicated (AUC 0.73) and high‐risk definitions (AUC 0.73) for the prediction of adverse events, while the fulminant classification showed significantly inferior predictive accuracy (AUC 0.62, *p* = 0.02). Moreover, among patients with preserved LVEF (≥50%) at presentation, NLR showed superior prognostic value (AUC 0.73; complicated: 0.52, *p* = 0.001; high‐risk: 0.52, *p* = 0.002). Multivariable analysis confirmed that each classification was independently associated with the primary outcome. However, the NLR model showed better predictive performance compared to the other models among patients with LVEF ≥50%.

**Conclusions:**

While traditional definitions of high‐risk AM remain valuable, NLR ≥4 is a simple and cost‐effective marker that aids in risk stratification. NLR ≥4 is particularly robust in patients with preserved LVEF, supporting its use across the LVEF spectrum.

## Introduction

Risk stratification in acute myocarditis (AM) is challenging as both the aetiology and clinical presentation are highly heterogeneous.^1^, ^2^, ^3^, ^4^, ^5^ Differences in diagnostic criteria, ranging from clinical suspicion to confirmation by cardiac magnetic resonance (CMR) imaging or endomyocardial biopsy (EMB), further impact risk assessment and prognostic accuracy.^2^ Current risk stratification models rely primarily on clinical parameters that are often not immediately available, such as left ventricular ejection fraction (LVEF), or reflect the clinical course during hospitalization, such as the need for inotropic or mechanical support.^6^, ^7^ Several classification models have been used to identify patients at increased risk of adverse outcomes, including ‘complicated’, ‘fulminant’ and ‘high‐risk’ AM (*Graphical Abstract*). However, these are typically applicable to patients with severe clinical manifestations or reduced LVEF at presentation.^1^, ^3^, ^8^, ^9^, ^10^ Conversely, due to the use of LVEF in most models, these are not applicable to patients with preserved or mildly reduced LVEF. Nonetheless, there is growing evidence that adverse events can still occur in patients conventionally considered at low risk.^10^, ^11^, ^12^ Furthermore, established classification models often rely on parameters assessed post‐acutely, limiting their utility for early risk stratification. This highlights a critical gap in stratifying risk across the full spectrum of AM presentations.

The neutrophil‐to‐lymphocyte ratio (NLR) is a non‐specific, inexpensive, and readily available biomarker in patients with AM. A cut‐off value of 4 has been recently associated with a worse prognosis in AM.^11^, ^13^, ^14^ However, the relative prognostic value compared to conventional high‐risk definitions remains unknown.

This study aimed to compare the prognostic value of available AM risk stratification models, including NLR ≥4, in a large cohort of patients with AM.

## Methods

### Study design

The design of this study has been previously described.^11^ Briefly, we included all consecutive patients aged ≥18 years admitted to 10 hospitals in six countries with a confirmed diagnosis of AM by either EMB or CMR.^11^ Patients presented with cardiac symptoms, significant elevation of troponin levels, and CMR findings consistent with AM according to 2018 updated Lake Louise criteria, in the absence of significant coronary artery disease (CAD) on invasive or non‐invasive coronary imaging or low likelihood of CAD.^6^, ^15^, ^16^ Patients with suspected/confirmed COVID‐19 or vaccine‐related AM were excluded from this analysis.^17^ Laboratory parameters at admission, echocardiography and CMR findings were recorded. EMB was performed according to centre‐specific criteria in suspected high‐risk cases.^7^ Patients were retrospectively classified using conventional models of AM as being at high‐risk of cardiovascular events that may require urgent and more intensive management, referred to as ‘complicated’, ‘fulminant’, ‘high‐risk’, and ‘NLR ≥4’.^8^, ^9^, ^10^, ^11^ Models of high‐risk AM are described in online supplementary *Table* *S1*. Models of high‐risk AM are not mutually exclusive due to overlapping diagnostic criteria. The study was conducted under London South‐East Research Ethics Committee approval (reference 18/LO/2048) granted to the King's Electronic Records Research Interface (KERRI) and local approval where needed. The study complied with the Declaration of Helsinki.

### Study outcome

The primary outcome measure was a composite of death or heart transplantation.

### Subgroup and sensitivity analysis

Pre‐specified subgroup analyses were performed to ascertain the usefulness of NLR in patients at low baseline risk indicated by an LVEF ≥50%, and in patients without autoimmune disease who are less likely to have a history of immunosuppression, and in patients who did not receive in‐hospital steroids to avoid potential confounding due to immunosuppressive therapy.

### Statistical analysis

Continuous variables were expressed as medians and interquartile ranges (IQR). Categorical variables were expressed as counts and percentages. Comparisons between groups were made by the Mann–Whitney U test for continuous variables or the Pearson's Chi‐square test or the Fisher's exact tests for discrete variables. NLR was analysed as a continuous variable in receiver operating characteristic (ROC) curve analyses to preserve the granularity of the data and improve categorization, while a previously validated cut‐off (NLR ≥4) was used in the regression models.^11^ ROC curves were calculated, and the area under the ROC curve (AUC) with 95% confidence interval (CI) was used to compare the ability of the definition of AM to predict the primary outcomes. Differences in AUC values between classification models were statistically compared using DeLong's test, with NLR serving as the reference model for all pairwise comparisons. Survival curves for the primary outcome were estimated and compared between groups using the log‐rank test. Univariable and multivariable Cox proportional hazard models were fitted to obtain hazard ratios (HR) for outcomes in the study population using clinically relevant variables at baseline. Model performance was further evaluated using the Akaike information criterion (AIC) and Bayesian information criterion (BIC), with lower values indicating better model fit. A *p*‐value of <0.05 was considered statistically significant. All analyses were performed with R statistical package version 4.4.2 (R Foundation, Vienna, Austria, https://www.r‐project.org/).

## Results

### Study population and baseline characteristics

**Figure 1:**
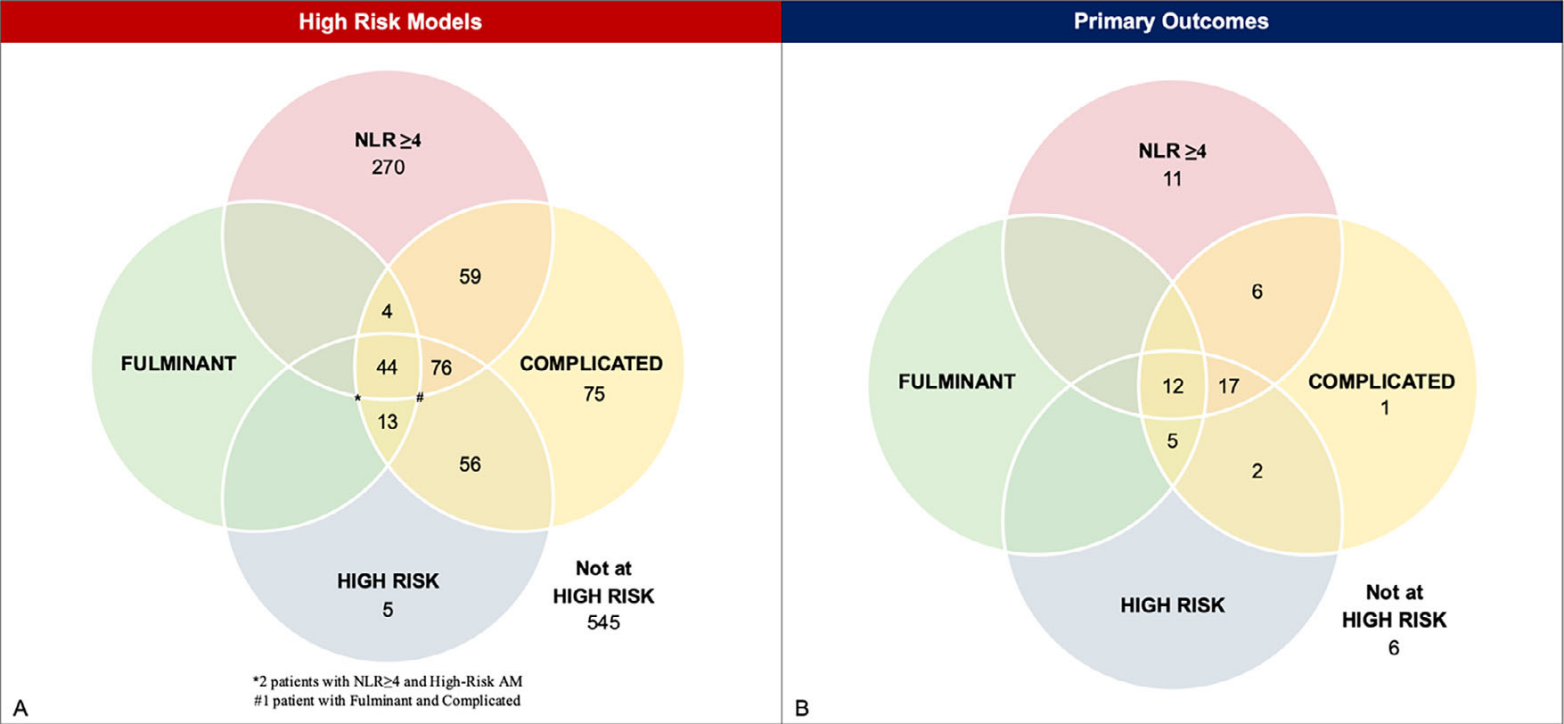
(*A*) Number of patients classified as high‐risk according to the four different definitions: complicated presentation, fulminant presentation, high‐risk, and neutrophil‐to‐lymphocyte ratio (NLR) ≥4. Patients may meet more than one high‐risk criterion, and overlaps are illustrated in the Venn diagram. (*B*) Distribution of patients who experienced the primary outcome, indicating how many events were correctly identified by each high‐risk definition. Overlaps represent patients who met more than one high‐risk classification among those who experienced an event.

A total of 1150 patients with CMR‐ or EMB‐proven AM were included in the study. Approximately two‐thirds of patients were male (*n* = 827, 72%) with a median age of 38 (IQR 26–50) years (*Table* *1*). Among them, 605 patients (53%) were considered at increased risk of adverse outcomes according to one or more existing models. A total of 328 (29%) patients were classified as having complicated AM, 62 (5%) as fulminant AM, 196 (17%) as high‐risk AM, and 455 (40%) as having NLR ≥4 (*Figure* *1*, *Table* *1*). Forty‐four (4%) patients met all four high‐risk classification models (*Figure* *1*). Among the 1150 patients analysed, 545 (47%) did not meet any of the classification model criteria and were therefore considered not at high‐risk.

**Table 1:** Baseline characteristics

	Total cohort	Complicated	Fulminant	High‐risk	NLR ≥4
Patients, *n*	1150	328	62	196	455
Male sex	827 (72)	205 (63)	33 (53)	115 (59)	312 (69)
Age (years)	38 [26–50]	45 [27–57]	46 [26–59]	48 [32–60]	41 [28–54]
Prodromal symptoms	700 (64)	173 (59)	36 (59)	93 (53)	289 (67)
Clinical presentation					
Chest pain	752 (76)	161 (59)	34 (56)	85 (52)	254 (67)
Dyspnoea	168 (17)	134 (49)	42 (69)	100 (61)	90 (24)
Arrhythmia	69 (7)	53 (20)	11 (18)	50 (31)	34 (9)
Previous myocarditis	30 (4)	10 (4.6)	1 (1.7)	4 (3)	6 (3)
Comorbidities					
Hypertension	134 (12)	50 (17)	9 (15)	30 (17)	64 (14)
Dyslipidaemia	135 (13)	38 (14)	9 (15)	24 (14)	69 (16)
Diabetes	56 (5)	22 (7.5)	4 (6.5)	12 (7)	27 (6)
CKD	23 (3)	14 (4.3)	1 (1.6)	11 (6)	15 (3)
Autoimmune disease	63 (9)	24 (7.3)	4 (6.5)	16 (8)	33 (7)
SBP	120 [109–130]	113 [100–124]	104 [92–120]	110 [100–123]	118 [105–130]
HR	84 [71–95]	90 [73–110]	110 [83–120]	97 [78–116]	89 [74–101]
Echo					
LVEF at admission (%)	52 [46–60]	39 [26–45]	26 [20–30]	30 [20–35]	48 [39–60]
LVSD	302 (28)	301 (92)	62 (100)	176 (90)	171 (38)
CMR					
LVEF (%)	57 [53–63]	53 [41–59]	53 [35–59]	51 [33–59]	56 [52–62]
LGE	872 (89)	198 (86)	24 (71)	98 (81)	312 (85)
In‐hospital management					
Inotropes	79 (7)	67 (21)	62 (100)	63 (34)	55 (12)
Steroids	188 (16)	105 (32)	33 (53)	79 (40)	97 (22)
Renal replacement therapy	23 (3)	18 (8.3)	11 (19)	16 (12)	17 (7)
Discharge medication					
Aspirin/NSAIDS	453 (41)	97 (33)	8 (13)	48 (28)	158 (37)
RAASi	428 (37)	193 (59)	40 (65)	124 (64)	180 (40)
Beta‐blockers	494 (43)	211 (65)	42 (68)	137 (70)	218 (48)
MRAs	106 (9)	78 (24)	14 (23)	54 (28)	47 (10)
Diuretics	131 (12)	97 (30)	31 (50)	76 (39)	60 (13)
Amiodarone	39 (6)	35 (18)	15 (25)	32 (26)	23 (10)
Immunosuppressants	132 (12)	84 (29)	24 (39)	62 (36)	79 (18)
Blood values					
CRP (mg/L)	62.5 [10–87]	48 [15–126]	73 [25–137]	41 [12–132]	91.7 [23–138]
Neutrophils (10∧9/L)	6.6 [4.1–8.2]	7 [4.9–10]	8.8 [7.2–14]	7.6 [5.4–10.9]	9 [6.9–11.0]
Lymphocytes (10∧9/L)	1.86 [1.29–2.20]	1.60 [1.2–2.1]	1.43 [1.1–1.81]	1.54 [1.1–2]	1.33 [0.96–1.62]
Monocytes (10∧9/L)	0.75 [0.46–0.91]	0.68 [0.43–0.91]	0.70 [0.45–0.91]	0.68 [0.42–0.92]	0.85 [0.52–1.05]
Eosinophils (10∧9/L)	0.10 [0.03–0.20]	0.08 [0.02–0.20]	0.05 [0.02–0.20]	0.06 [0.02–0.20]	0.06 [0.02–0.12]
Basophils (10∧9/L)	0.05 [0.02–0.06]	0.03 [0.01–0.08]	0.06 [0.01–0.10]	0.03 [0.01–0.09]	0.04 [0.01–0.05]
Endomyocardial biopsy	201 (18)	139 (42)	39 (63)	108 (55)	93 (21)

Values are reported as *n* (%), or median [25th–5th centiles].

CKD, chronic kidney disease; CRP, C‐reactive protein; HR, heart rate; LGE, late gadolinium enhancement; LVEF, left ventricular ejection fraction; LVSD, left ventricular systolic dysfunction; MRA, mineralocorticoid receptor antagonist; NLR, neutrophil‐to‐lymphocyte ratio; NSAID, non‐steroidal anti‐inflammatory drug; RAASi, renin–angiotensin–aldosterone system inhibitor; SBP, systolic blood pressure.

Patients with fulminant or high‐risk AM were generally older compared to other groups (*Table* *1*). The comorbidity profile was similar across the four classification groups. Patients with NLR ≥4 were less likely to have left ventricular systolic dysfunction (LVSD, defined as LVEF <50%) compared to the other groups (NLR ≥4 38%; complicated 92%; fulminant 100%; high‐risk 90%), and generally required less inotropic support (NLR ≥4 12%; complicated 21%; fulminant 100%; high‐risk 34%), steroid therapy (NLR ≥4 22%; complicated 32%; fulminant 53%; high‐risk 40%), and renal replacement therapy (NLR ≥4 7%; complicated 8%; fulminant 19%; high‐risk 12%) during hospitalization (*Table* *1*). C‐reactive protein levels varied across groups, with the highest median value observed in the NLR ≥4 group (NLR ≥4 91.7 mg/L [23–138]; complicated 48 mg/L [15–126]; fulminant 73 mg/L [25–137]; high‐risk 41 mg/L [12–132]) (*Table* *1*).

### Primary outcome

During a median follow‐up of 228 weeks [IQR 114–339], 63 events occurred in 60 patients (5.2%) who experienced the primary outcome. Among them, 48 patients (4.2%) died, and 15 patients (1.4%) underwent heart transplantation. Identification of patients who experienced adverse events varied according to different models. NLR ≥4 identified 46 (77%) patients who experienced the primary outcomes, while complicated AM identified 43 (72%) patients, fulminant AM identified 17 (28%) patients, and high‐risk AM identified 36 (60%) patients who experienced the primary outcome (*Figure* *1*).

**Figure 2:**
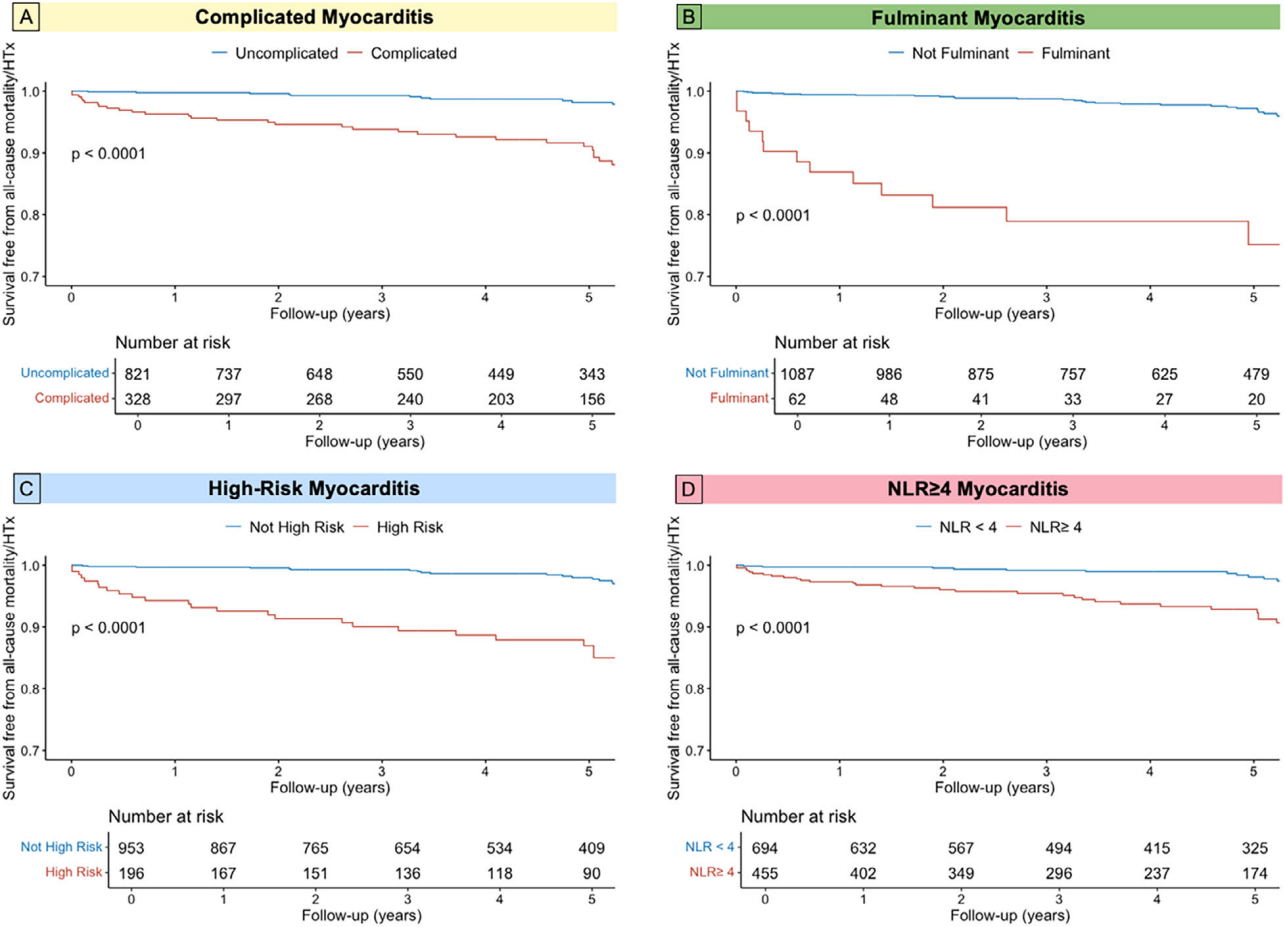
Kaplan–Meier curve for all‐cause mortality or heart transplantation (HTx) according to complicated myocarditis (*A*), fulminant myocarditis (*B*), high‐risk myocarditis (*C*), and neutrophil‐to‐lymphocyte ratio (NLR) ≥4 (*D*).

**Figure 3:**
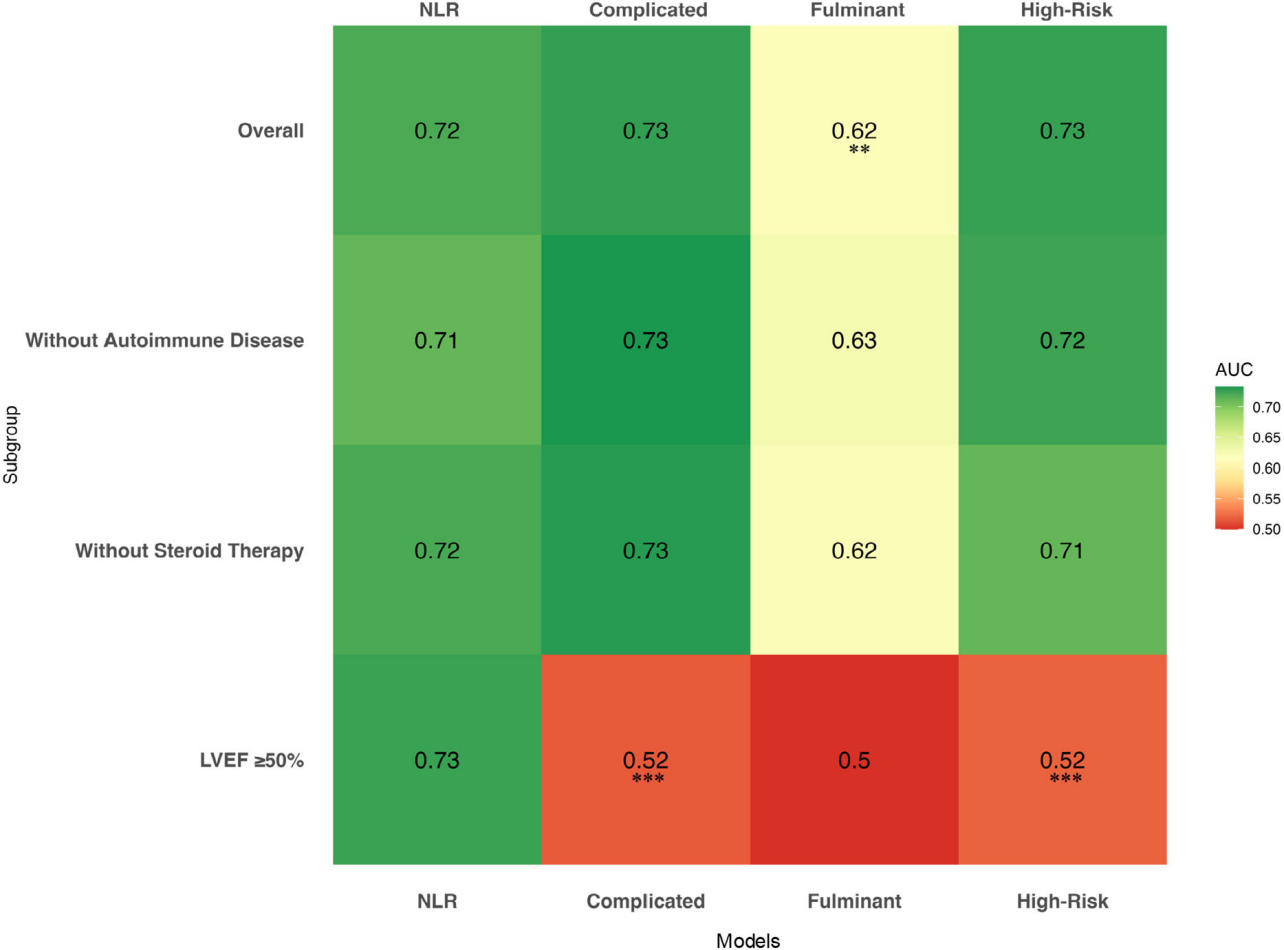
Area under the curve (AUC) for all‐cause mortality or heart transplantation according to neutrophil‐to‐lymphocyte ratio (NLR), complicated myocarditis, fulminant myocarditis, and high‐risk myocarditis in the overall population, in the subgroups of patients without autoimmune disease, without steroid therapy and with left ventricular ejection fraction (LVEF) ≥50%. **p < 0.05; ***p < 0.005.

All four high‐risk classification models were associated with an increased risk of adverse events (all *p* < 0.0001; *Figure* *2*). To assess the predictive value of the four models of high‐risk AM, we calculated the AUC for each model. Compared to NLR, complicated and high‐risk demonstrated similar discriminative ability, while the fulminant phenotype had a significantly lower AUC (AUC for NLR 0.72; AUC for complicated 0.73, *p* = 0.85 vs. NLR; AUC for fulminant 0.62, *p* = 0.023 vs. NLR; AUC for high‐risk 0.73, *p* = 0.88 vs. NLR; *Figure* *3*).

**Figure 4:**
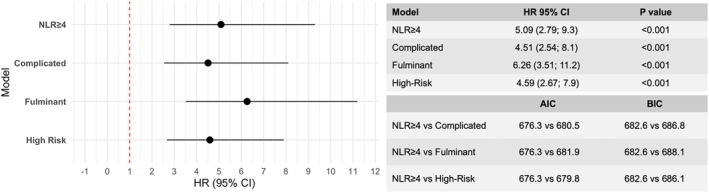
Forest plot of multivariable analysis showing the overall association of classification models with the primary outcomes in acute myocarditis patients, adjusted for sex and age. AIC, Akaike information criterion; BIC, Bayesian information criterion; CI, confidence interval; HR, hazard ratio; NLR, neutrophil‐to‐lymphocyte ratio.

All models remained associated with the study outcome in a multivariable analysis after adjustment for sex and age (NLR ≥4: HR 5.09, 95% CI 2.79–9.28; *p* < 0.001; complicated: HR 4.51, 95% CI 2.54–8.1; *p* < 0.001; fulminant: HR 6.26, 95% CI 3.51–11.2; *p* < 0.001; high‐risk: HR 4.59, 95% CI 2.67–7.9; *p* < 0.001) (*Figure* *4*). Furthermore, lower AIC and BIC values of the NLR ≥4 model compared to traditional high‐risk classification models indicate a better overall model fit, despite showing similar discriminative performance (*Figure* *4*).

### Subgroup and sensitivity analysis

Sensitivity analyses were performed to account for clinical scenarios traditionally associated with higher baseline risk, such as autoimmune conditions requiring immunosuppressive therapy and the use of steroids during hospitalization. Our findings remained consistent among patients without autoimmune disease (AUC for NLR: 0.71; complicated: 0.73, *p* = 0.62 vs. NLR; fulminant: 0.63, *p* = 0.07 vs. NLR; high‐risk: 0.72, *p* = 0.78 vs. NLR) and in those who did not receive steroid therapy in hospital (AUC for NLR: 0.72; complicated: 0.73, *p* = 0.86 vs. NLR; fulminant: 0.62, *p* = 0.09 vs. NLR; high‐risk: 0.71, *p* = 0.87 vs. NLR; *Figure* *3*).

**Figure 5:**
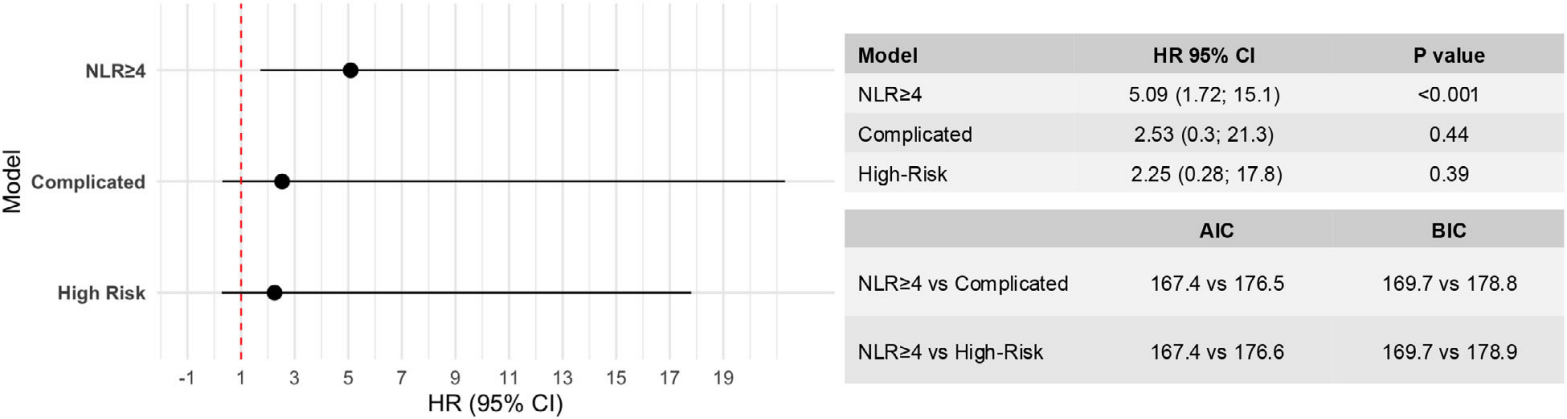
Forest plot of multivariable analysis showing the overall association of classification models with the primary outcomes in acute myocarditis patients with preserved left ventricular ejection fraction at baseline, adjusted for sex and age. AIC, Akaike information criterion; BIC, Bayesian information criterion; CI, confidence interval; HR, hazard ratio; NLR, neutrophil‐to‐lymphocyte ratio.

Among patients with preserved LVEF (≥50%), who are considered at low risk by existing models (the fulminant classification excluded patients with LVEF >50% and was excluded from this analysis), NLR was superior in identifying patients at risk of adverse events compared to the other definitions (AUC for NLR: 0.73; AUC for complicated: 0.52, *p* = 0.001 vs. NLR; AUC for high‐risk: 0.52, *p* = 0.002 vs. NLR; *Figure* *3*). After adjusting for age and sex, NLR≥4 was the strongest predictor of the primary outcome in patients with LVEF >50% (NLR ≥4: HR 5.09, 95% CI 1.72–15.1; *p* < 0.001; complicated: HR 2.53, 95% CI 0.3–21.3; *p* = 0.44; high‐risk: HR 2.25, 95% CI 0.28–17.8; *p* = 0.39) (*Figure* *5*). Among patients with preserved LVEF, NLR ≥4 demonstrated the best performance in predicting the primary outcomes, as shown by the AIC and BIC values (*Figure* *5*).

## Discussion

Risk stratification in AM remains a clinical challenge, as the disease encompasses a broad spectrum of presentations and outcomes. Current classification models, including complicated, fulminant and high‐risk, provide valuable insights for identifying patients at risk of adverse events.^1^, ^8^, ^9^, ^10^, ^18^, ^19^ These models have predominantly identified severe clinical manifestations as high‐risk features, including arrhythmia or significantly reduced LVEF at presentation. However, more subtle predictors may have been overlooked due to limited statistical power, thereby limiting their applicability to the broader AM population. We previously reported that NLR ≥4 is associated with adverse outcomes across the spectrum of baseline LVEF in the largest reported cohort of CMR‐ or EMB‐proven AM.^11^ Here, we compared NLR to other available classification models.

The characteristics of our study population are similar to those of the cohorts used to develop previous classification models, enabling valid comparisons. In this study, we report that NLR is comparable to established high‐risk models in predicting mortality and heart transplantation.

Importantly, in patients with preserved LVEF (≥50%), a subgroup often excluded from established risk models, NLR emerged as the only model capable of reliably identifying individuals at increased risk of adverse outcomes since it does not include reduced LVEF or haemodynamic compromise variables. This reinforces the notion that NLR may be a reliable risk indicator across the entire spectrum of AM. This finding is particularly relevant considering that LVEF is a late‐stage indicator of disease severity, often remaining within normal range until advanced stages or overt haemodynamic compromise.^20^, ^21^ In contrast, NLR reflects early inflammatory activity, capturing biology before mechanical dysfunction is established.

The NLR has gained increasing attention as a marker of systemic inflammation and immune activation in various cardiovascular diseases.^13^, ^14^, ^22^ In our study, patients with NLR ≥4 had higher C‐reactive protein values, identifying a subgroup with higher systemic inflammation. Therefore, elevated NLR values may reflect an injurious innate immune response driven by enhanced neutrophil egress from the bone marrow and inflammation, although further studies are required to characterize this.^4^, ^23^ Notably, after excluding patients with autoimmune diseases or those who had received steroid therapy, which are potential sources of increased inflammation and elevated NLR, the results remained consistent. Other novel biomarkers, such as non‐coding circulating RNAs, are expensive, not widely available, and require longer processing times, limiting their practical application.^15^, ^24^ Furthermore, their role in prognostic stratification remains poorly understood.^9^, ^10^ NLR is inexpensive and easy to measure. It could therefore serve as a pragmatic tool for early risk stratification in AM, providing an initial and accurate assessment of the patient as soon as blood tests are evaluated in the emergency department, even before imaging or EMB are considered. This makes NLR particularly valuable in settings where access to CMR or EMB is limited, especially in low‐ and middle‐income countries where myocarditis is more prevalent but diagnostic resources are often scarce.^25^

Models of complicated, fulminant, and high‐risk myocarditis are based not only on clinical presentation, but also on therapeutic interventions such as the requirement for inotropic support and LVEF assessment, limiting their use in early risk stratification and introducing bias.^9^, ^15^, ^26^ Even in the case of giant cell myocarditis (GCM), although rare, giant cells may take up to 2 weeks to appear in histological samples, leading to delays in diagnosis and disease classification.^27^ In contrast, simple blood tests are typically performed and reported immediately in patients with suspected AM to assess biomarkers such as troponin and C‐reactive protein.^28^

The findings of our study have important clinical implications. First, they suggest that NLR can be considered as part of routine risk assessment in patients with AM. Second, its ability to identify high‐risk patients among those with preserved LVEF challenges the current paradigm that associates risk primarily with left ventricular dysfunction. In clinical practice, this could assist clinicians in identifying high‐risk patients earlier in the disease course, allowing for closer monitoring and follow‐up in specialist myocarditis services for those with AM who might otherwise be considered low risk (*Graphical Abstract*). Moreover, given the widespread availability of NLR, incorporating it into clinical decision‐making may be especially beneficial in resource‐limited settings, where access to advanced imaging or biopsy is restricted.

Although the primary objective of our study was to evaluate prognostic modelling among AM patients, the implications of risk stratification on therapeutic strategies also requires consideration.^29^ The role of immunosuppressive therapy in AM remains the subject of several ongoing studies.^29^ While corticosteroids have been used in selected cases, particularly in eosinophilic AM and GCM, their efficacy in broader AM populations is still debated. The Myocarditis Treatment Trial randomized 111 patients with a histopathological diagnosis of myocarditis and LVEF <45% to receive prednisone and cyclosporine or azathioprine in association with standard therapy versus standard therapy alone.^30^ The primary outcome was mean change in LVEF at 28 weeks, which did not differ significantly between patients who received immunosuppressive therapy (a gain of 0.10%; 95% CI 0.07–0.12%) and the control group (a gain of 0.07%; 95% CI 0.03–0.12%).^30^ The MYocarditis Therapy with Steroids (MYTHS) study is currently exploring the safety and efficacy of empirical high‐dose corticosteroid therapy to treat patients with complicated/fulminant AM.^31^ It may also be feasible to use NLR as a biomarker to identify patients who could benefit from corticosteroids but may hitherto have been excluded from clinical trials.

The NLR may also help identify patients who could most benefit from other immunosuppressive treatments. Interleukin‐1β (IL‐1β) antagonists such as anakinra have emerged as potential treatment options and have been shown to reduce NLR values in cardiovascular disease.^32^ The Anakinra versus placebo double blind Randomized controlled trial for the treatment of Acute MyocarditIS (ARAMIS) trial was the first randomized study evaluating the inhibition of IL‐1β in AM.^33^ The trial did not demonstrate a significant reduction in its primary endpoint with anakinra, compared to placebo, which may reflect a lack of patient stratification. Prospective randomized studies incorporating NLR as an inclusion criterion are needed to validate its role in guiding therapy and improving clinical outcomes in AM patients.

### Limitations

This analysis is conducted on the largest international cohort of consecutive patients with confirmed AM reported so far. However, potential selection and referral bias cannot be excluded, and the role of unmeasured confounders may limit the generalizability to all AM patients. Aetiology of AM, data on the genetic background and on mechanical circulatory support were not available for all patients. In patients with available CMR data, the precise localization of late gadolinium enhancement was not consistently reported, limiting the ability to refine the definition of complicated AM.^34^ Given the available data, it was not possible to investigate dynamic changes of biomarkers and LVEF, both acutely or over follow‐up. Although several factors might influence NLR, including administration of steroids and other immune modulators, and viral and bacterial infections, we did not have sufficient power to account for this, and the observational nature of our study did not allow us to infer causality.

## Conclusion

A NLR ≥4 is a strong and independent marker for identifying high‐risk patients with AM, which offers a simple and widely accessible risk stratification model. Its performance in patients with preserved LVEF suggests that NLR could also enhance risk stratification across the spectrum of AM patients. These findings support the integration of NLR into clinical decision‐making, offering a pragmatic approach to identify high‐risk patients early and potentially improve outcomes in AM.

### Funding

A.C. is supported by the British Heart Foundation (FS/CRTF/21/24175). A.M.S. is supported by the British Heart Foundation (CH/1999001/11735). D.B. is supported by a Medical Research Council Clinician Scientist Fellowship (MR/X001881/1) and the King's British Heart Foundation Centre of Research Excellence (RE/18/2/34213). The funders played no role in the study design, collection, analysis, interpretation of data, writing of the report, or in the decision to submit the paper for publication. They accept no responsibility for the contents.

**Conflict of interest**: C.T. has received speaker fees and/or contributions to meetings from Abbott, Abiomed, AstraZeneca, Bayer, BMS, Boston Scientific, Impulse Dynamics, Novartis, Pfizer, MS, and Viofor, all outside of the submitted work. C.G. has received research funding from the Swiss National Science Foundation and Innosuisse, the Center for Artificial Intelligence in Medicine Research Project Fund University Bern, Novartis Science Foundation and the GAMBIT Foundation, outside of the submitted work. P.A. has received speaker and/or consultancy fees from AstraZeneca, Boehringer Ingelheim, Bayer, Novartis, Daiichi Sankyo, Janssen and MSD, all outside the submitted work. All other authors have nothing to disclose.

## Supporting information


**Appendix S1.** Supporting Information.
