# Supplementary material for: Neutrophil‐to‐lymphocyte ratio for risk stratification in acute myocarditis across the left ventricular ejection fraction spectrum
Source: Eur J Heart Fail. 2025 Oct 16;27(12):3278–87. doi: 10.1002/ejhf.70072 (PMC12803673; doi:10.1002/ejhf.70072)
Supplement: Supplementary file 1 — Appendix S1. Supporting Information. [file EJHF-27-3278-s001.docx]

**Supplementary Table 1.** Published models of AM with higher risk of adverse outcomes

| Complicated Myocarditis | Fulminant Myocarditis | High Risk  Myocarditis | Neutrophil-Lymphocyte ratio (NLR) ≥4 Myocarditis |
| --- | --- | --- | --- |
| Presence of ≥1 critical features, such as LVEF <50% on the first in-hospital echocardiogram, sustained ventricular arrhythmias (SVA), complete atrioventricular block (AVB), or with fulminant presentations (10). | Severe, rapidly evolving form of the disease characterized by significant hemodynamic compromise requiring inotropes and/or mechanical circulatory support (8). | Decompensated heart failure, severe LVSD (with an LVEF below 40%), life-threatening arrhythmias regardless of the degree of LV dysfunction, and advanced AVB when associated with LV dysfunction (1,9). | A value ≥4 at the first available complete blood count after admission (11). |
